# Assessing the external validity of model-based estimates of the incidence of heart attack in England: a modelling study

**DOI:** 10.1186/s12889-016-3782-6

**Published:** 2016-11-03

**Authors:** Peter Scarborough, Kate Smolina, Anja Mizdrak, Linda Cobiac, Adam Briggs

**Affiliations:** 1 British Heart Foundation Centre for Population Approaches to Non-Communicable Disease Prevention, Nuffield Department of Population Health, University of Oxford, Oxford, UK; 2 British Columbia Centre for Disease Control, Vancouver, Canada; 3 Burden of Disease Epidemiology, Equity and Cost Effectiveness (BODE3) Programme, University of Otago, Wellington, New Zealand

**Keywords:** Myocardial infarction, Incidence, Validity, Modelling, DisMod

## Abstract

**Background:**

The DisMod II model is designed to estimate epidemiological parameters on diseases where measured data are incomplete and has been used to provide estimates of disease incidence for the Global Burden of Disease study. We assessed the external validity of the DisMod II model by comparing modelled estimates of the incidence of first acute myocardial infarction (AMI) in England in 2010 with estimates derived from a linked dataset of hospital records and death certificates.

**Methods:**

Inputs for DisMod II were prevalence rates of ever having had an AMI taken from a population health survey, total mortality rates and AMI mortality rates taken from death certificates. By definition, remission rates were zero. We estimated first AMI incidence in an external dataset from England in 2010 using a linked dataset including all hospital admissions and death certificates since 1998. 95 % confidence intervals were derived around estimates from the external dataset and DisMod II estimates based on sampling variance and reported uncertainty in prevalence estimates respectively.

**Results:**

Estimates of the incidence rate for the whole population were higher in the DisMod II results than the external dataset (+54 % for men and +26 % for women). Age-specific results showed that the DisMod II results over-estimated incidence for all but the oldest age groups. Confidence intervals for the DisMod II and external dataset estimates did not overlap for most age groups.

**Conclusion:**

By comparison with AMI incidence rates in England, DisMod II did not achieve external validity for age-specific incidence rates, but did provide global estimates of incidence that are of similar magnitude to measured estimates. The model should be used with caution when estimating age-specific incidence rates.

## Background

For many diseases, estimates of incidence and prevalence are often incomplete or based on different data sources making it difficult to compare results [1]. Reliable and representative population level epidemiological data are needed to inform health care policy and to support decision making processes in health service planning and delivery, and are essential to cost effectiveness analyses and burden of disease calculations. Due to gaps in directly measured data, models have been established that can estimate incidence and prevalence rates of diseases. DisMod II is such a model. It uses available epidemiological data about a condition to estimate missing data on incidence, prevalence, remission and case fatality rates as applicable [2, 3]. Originally developed for the Global Burden of Disease studies [4], DisMod II is freely available for use and can be downloaded from http://www.epigear.com/index.htm.

The DisMod II model is a multistate life table that fully describes the epidemiological progress of a single disease by exploiting the fact that parameters such as incidence, prevalence, remission, case fatality and mortality rates are not independent variables. By solving a set of differential equations, Dismod II can estimate age-specific incidence, prevalence or case fatality rates for a disease, given sufficient data on the other (for example, with input data of age-specific prevalence, case fatality and mortality data for a disease, Dismod II will estimate the age-specific incidence rate for the disease). The model operates by calculating the number of people in each of three states: healthy, diseased and dead at any age. Within the model, there are two causes of death, either from the disease or from ‘all other’ causes, that are assumed to be independent. There are four transition hazards which are age specific (assumed to be constant within a 1-year age interval): incidence, remission, case fatality, and the “all other mortality” hazard. The input data for the model are age and sex-specific estimates of three out of the four parameters described above for a given population, and a complete set of parameters (smoothed from the original or estimated from the original parameters) is the output of the model.

Ischaemic heart disease (IHD) is the most common cause of death in the UK [5] and acute myocardial infarction (AMI) is coded on death certificates as the cause of approximately one third of all deaths from IHD [6]. AMI mortality and prevalence data for having had an AMI in England are routinely collected by the Office of National Statistics (ONS) and the Health Survey for England (HSfE) [7] respectively. However, until recently, there have been no published comprehensive, population-based national level estimates of AMI incidence [8] and these recent estimates are unlikely to be routinely updated. Incidence of AMI is important to researchers and public health policy makers because it serves as an indicator of the effectiveness of preventative measures and management of risk factors through health promotion and other public health initiatives. Without a tool that allows routinely updated estimates of incidence, measurements of the current burden of AMI in England have significant limitations.

This study assesses the external validity of DisMod II estimates of the incidence of first AMI in England by comparison with estimates generated from the dataset used to support a recent series of related papers [8–10]. Establishing the external validity of the modelled estimates would demonstrate that the DisMod II model could be used as a tool for regularly updating estimates of the incidence of AMI—data that are not routinely collected in England. It would also help to establish confidence in studies of non-communicable disease that use DisMod II to estimate incidence, such as in modelling studies [11], and studies estimating disease burden where incidence data are scarce or not regularly updated [12, 13]. Since the 2010 iteration, the Global Burden of Disease study [4] results have been based on an updated (but closely related) version of the DisMod model, which is not freely available for use. Assessments of the external validity of DisMod II offer insights into the assumptions used for this important and widely used global project.

## Methods

### Disease definitions

The exact definitions that were used in this paper for the condition under investigation are provided in Table 1. In this table, the model outcome that was compared with the external datasets is prefixed with OUTCOME. The remaining model definitions are descriptions of the theoretical measures that are consistent with the outcome of interest, and the corresponding external data definitions describe where the model input data were taken from, and how similar they are to the theoretical measures.

**Table 1:** Model definitions, input data sources and external data sources

	Model definition (relevant year in brackets)	External data definition (data source in brackets)
Incidence	OUTCOME: Incidence of first AMI (2010)	Incidence of first AMI since 1998 (linked hospital episodes and mortality statistics, 2010).
Prevalence	Prevalence of ever having had an AMI (2010)	Prevalence of ever having had a doctor-diagnosed AMI (Health Survey for England, 2011).
Remission	Zero^a^	Zero
Excess mortality	Excess mortality due to first AMI (2010)	Death where AMI is included anywhere on the death certificate (ONS mortality statistics, 2010).

*Abbreviations*: *ONS* Office for National Statistics, *AMI* Acute Myocardial Infarction

^a^ Remission is zero because the prevalence data measures people who have ever been diagnosed with AMI

## Outcome measures

For AMI, *measured* data on prevalence, excess mortality and remission were used to create *modelled* data on incidence. The modelled incidence data were compared against *measured* estimates from the dataset used for similar results published in the peer reviewed literature which were independent of all input data to the DisMod II model [8]. The difference between the modelled and the measured estimates was calculated and displayed for all ages. Differences were recorded in percentages, with the measured data as the baseline.

## Data sources

### Model inputs

Single-year population estimates by sex were taken from the Office for National Statistics (ONS), for the year 2010. Mortality data where AMI was recorded as the underlying or contributory cause of death (ICD-10 codes I21-I22) by sex and 5 year age groups were provided by ONS. Part of the modelling process for DisMod II is to interpolate data into single year estimates, in order to have smooth rates for all the consistent modelled outputs. For the mortality data, this was achieved using a cubic spline interpolation on a log transformation of the original data.

**Fig. 1:**
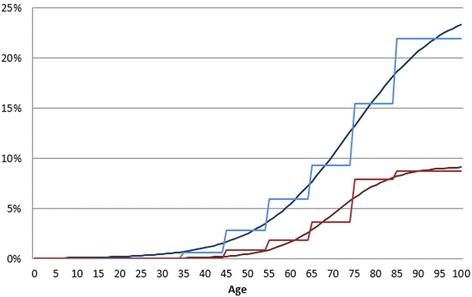
The data inputs used for the modelling exercise: prevalence of acute myocardial infarction by age and sex, actual data and smoothed data. The blue lines show actual and smoothed data for men and the red lines show actual and smoothed data for women

**Fig. 2:**
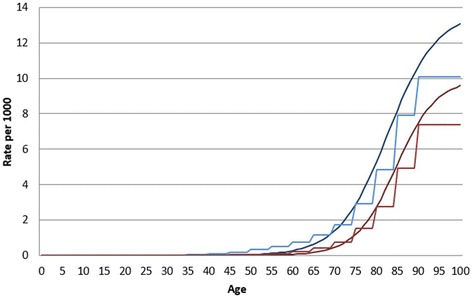
The data inputs used for the modelling exercise: excess mortality rates for acute myocardial infarction, actual data and smoothed data. The blue lines show actual and smoothed data for men and the red lines show actual and smoothed data for women

Age and sex-specific estimates of the prevalence of ever having had a doctor’s diagnosis of heart attack were taken from the Health Survey for England (HSfE) 2011 [7]. The HSfE covers all of England and is a nationally representative sample of those residing at private residential addresses. In the 2011 survey, a sample of 8610 individuals between aged 16 and 99 was recruited, with a household response rate of 66 %. Residents of care homes, prisons and the homeless were excluded which is thought to contribute to <2 % of the population. Those who were unable to consent and those who were unable to understand the questions or formulate answers (due to language difficulties, disability or mental illness) were termed non-responders. Figures 1 and 2 show the model input data (both raw and smoothed) by age for men and women.

### External validation

A recent series of papers examined AMI incidence, case fatality, survival, and trends in event rate, case fatality and mortality between 2002 and 2010 in a population based study using person linked routine hospital and mortality data in England [8–10]. Hospital episode statistics provide information on all patients admitted to hospital whose care is funded by the English NHS. An AMI event was classified as an emergency hospital admission with primary diagnosis of AMI and a length of stay of more than 1 day for someone discharged alive, or a death with acute myocardial infarction coded as the underlying cause of death on the death certificate. Fatal cases were defined as those where AMI was coded as the underlying cause of death on the death certificate or any death that occurred within 30 days of an admission for AMI which was assumed to relate to the same event. Bespoke analyses using the same dataset and methods have been used to generate measures of the incidence of first AMI in England in 2010, which are used as the external comparator in these analyses. Because the linked dataset used for these analyses only includes hospital episodes and deaths from 1998 onwards, these measures are strictly of the incidence of first AMI *since 1998* in England in 2010 (i.e. a person who had a first heart attack before 1998 and then a second heart attack in 2010 would be counted as a first heart attack in our dataset).

## DisMod II settings

Prevalence, remission (set as zero), and disease specific mortality were used as inputs in the DisMod II model. Population numbers and mortality rates for England 2010 were also used. The prevalence and mortality data were fitted with a sigmoid mathematical curve in order to smooth out the data points which have relatively large age group widths. Analyses were conducted with and without accounting for trends in AMI incidence rates. When trends were applied, an annual change in incidence rate of −4 % and an annual change in case fatality of −1 % for the previous 28 years was incorporated into the model. These trends are based on a study of trends in CHD since 1978 [14]. For a sensitivity analysis, larger trends were also applied that were taken from an analysis of incidence and case fatality rates over the previous 10 years in England [9]—these were −5 % for incidence and −4 % for case-fatality for both men and women. Application of a trend in DisMod II assumes that each age group is changing at the same rate as the overall trend. The DisMod II uncertainty analysis was conducted to assess uncertainty around the modelled incidence estimates. Here, DisMod II conducts a parametric bootstrapping exercise, where the input data are allowed to vary according to a specified distribution. We allowed the estimates of prevalence of having had a heart attack to vary with a normal distribution according to the uncertainty reported in the HSfE. We did not specify any uncertainty in either the mortality or the remission estimates. Due to lack of computing power, it was not possible to conduct an uncertainty analysis for the DisMod II runs where trends were assumed.

The complete set of results and the data used to run the DisMod II model for these analyses are available from the authors upon request.

## Results

**Fig. 3:**
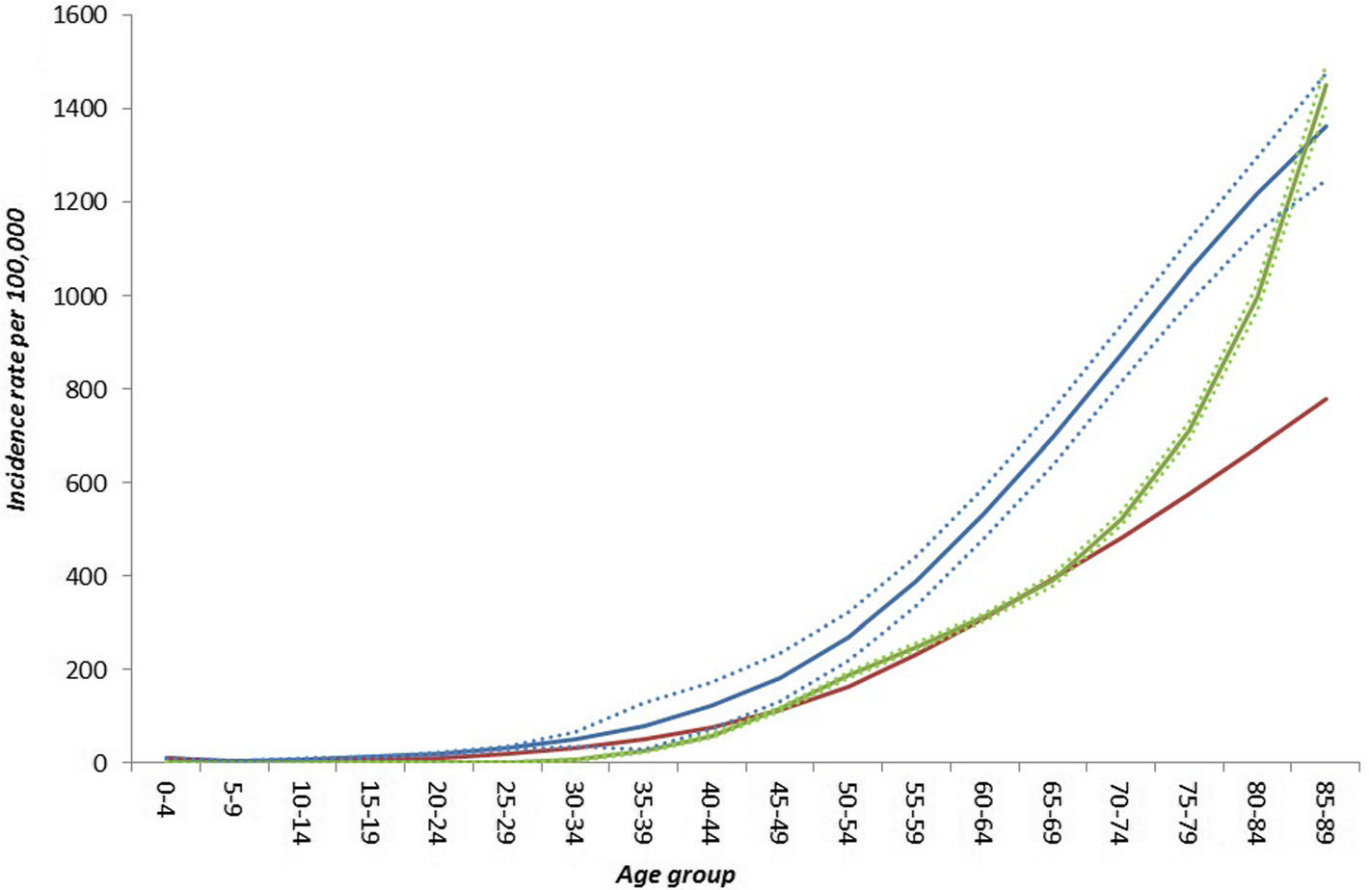
The incidence rate per 100,000 of first acute myocardial infarction in males in England in 2010. The green lines are estimates from the external dataset with 95 % confidence intervals. The blue lines are estimates from DisMod II with 95 % credible intervals that do not account for trends in incidence and case fatality. The red line is the estimate from DisMod II that does account for trends in incidence and case fatality

**Fig. 4:**
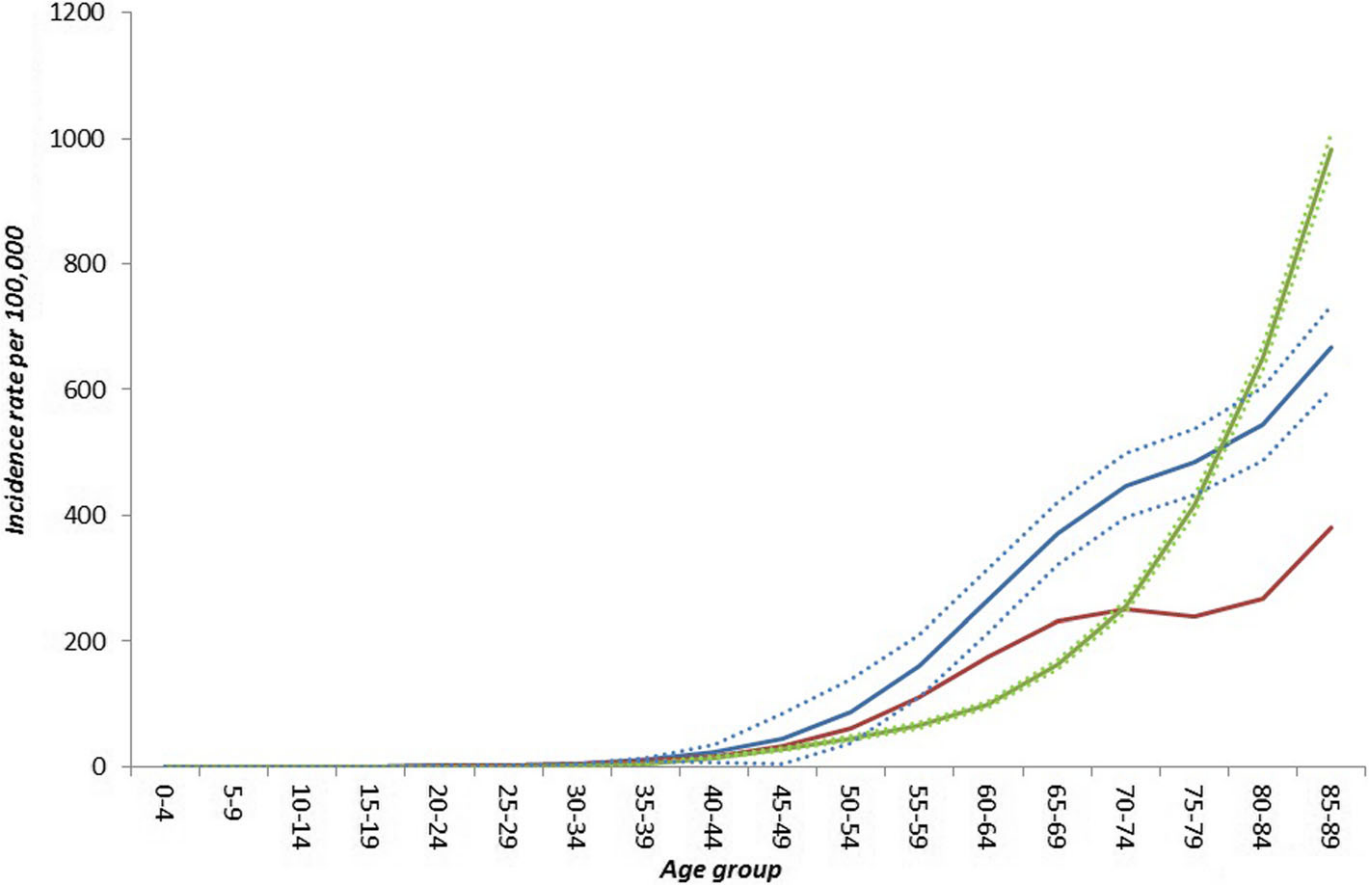
The incidence rate per 100,000 of first acute myocardial infarction in females in England in 2010. The green lines are estimates from the external dataset with 95 % confidence intervals. The blue lines are estimates from DisMod II with 95 % credible intervals that do not account for trends in incidence and case fatality. The red line is the estimate from DisMod II that does account for trends in incidence and case fatality

Figures 3 and 4 show the age-specific estimates of incidence of AMI for men and women separately. The green lines show the estimates from the external dataset with accompanying 95 % confidence intervals and the blue lines show the estimates from DisMod II with accompanying 95 % credible intervals (i.e. the distance between which 95 % of the iterations of the uncertainty analysis fell). The red line shows the estimates from DisMod II where trends in incidence and case fatality have been incorporated. The figures show that, for both men and women, the DisMod II estimates tend to over-estimate the incidence of AMI at younger age groups and under-estimate for the oldest age groups (greater than 85 in men, and greater than 75 in women). For both men and women, for the majority of age groups the confidence intervals between the external dataset and the DisMod II estimates did not overlap, suggesting poor external validity of the DisMod II estimates as AMI incidence rates increase. Applying trends in incidence and case fatality rates to the DisMod II estimates resulted in reduced incidence rates at all ages than without adjustment and an s-shaped curve for AMI incidence in women where the model estimated a lower incidence in women in their 70s compared to those in their 60s. The sensitivity analysis with larger trends in incidence resulted in further reductions in the estimates of MI incidence for both men and women, but the s-shaped curve in women did not persist (data not shown). Table 2 shows how the non-trended DisMod II estimates of incidence rates for the total population produced over-estimates of the incidence of MI by approximately 54 % in men and 26 % in women, but this masks larger differences for age-specific subgroups, including under-estimates of incidence in the oldest age groups.

**Table 2:** Number of first AMI events in England, 2010

	Number		Incidence rate per 100,000 (and 95 % confidence intervals)		
	External Dataset	DisMod II^a^	External Dataset	DisMod II^a^	% difference
Men					
0–29	105	1588	1 (1, 1)	16	1412
30–54	7035	12,558	79 (77, 81)	141	79
55–64	8446	13,919	282 (276, 288)	465	65
65–74	9674	16,703	451 (442, 460)	779	73
75–84	10,451	14,147	828 (812, 844)	1121	35
85+	5978	5487	1521 (1483, 1559)	1396	−8
Total	41,689	64,401	162 (160, 163)	250	54
Women					
0–29	38	88	0 (0, 1)	1	132
30–54	1752	3091	19 (19, 20)	34	76
55–64	2573	6664	83 (79, 86)	214	159
65–74	4817	9534	205 (200, 211)	407	98
75–84	8640	8490	521 (510, 532)	512	−2
85+	8911	5903	1107 (1084, 1130)	733	−34
Total	26,731	33,769	101 (100, 102)	128	26

^a^The DisMod II results are those estimated without the application of trend data

## Discussion

This study assessed the external validity of DisMod II estimates of the age-specific incidence rate of AMI in England in 2010. Although the modelled estimates and the external dataset resulted in incidence rates in the whole population that were of similar magnitude, the age-specific rates were not consistent with the external dataset; they over-estimated rates in younger age groups and under-estimated rates in the oldest age groups. Incorporating trends in the incidence and case fatality of AMI in the DisMod II estimates resulted in estimated and observed AMI incidence being more closely matched at younger age groups than without trends but with more divergent results at older ages, including for one set of results the implausible scenario of women in their 70s having a lower incidence of AMI than women in their 60s. This study implies that DisMod II is not an appropriate source of age-specific estimates of the incidence of AMI for England and future estimates should be based on measured outcomes.

Modelled estimates of the burden of disease in different populations are extremely important for policy makers and health care planners. They allow for an assessment of where to direct scarce resources in terms of treatment and care, and assist in planning for future health care requirements. They can also be used as the basis for comparing the burden of disease that is attributable to different behavioural risk factors (e.g. the global comparable risk assessment exercise, Global Burden of Disease [3, 15], or modelling studies to assess the effectiveness or cost-effectiveness of public health interventions [11, 16, 17] which in turn influences how public health resources are directed).

The DisMod II model is the most recent freely available version of the model that is used for the Global Burden of Disease project and it is currently being used by non-communicable disease scenario models [18] which have been built in order to estimate the population health impact of public health interventions. For example, Cecchini et al. (2010) [11] used DisMod II to estimate the incidence of various cardiovascular diseases and risk factors when simulating the possible effects on health of different diet and physical activity interventions. Previous studies have also used DisMod II to estimate disease incidence from prevalence, remission, and disease specific mortality rates where incidence data are scarce. For example, Johnston et al. (2009) [13] estimated the global stroke burden and used DisMod II to estimate stroke incidence from mortality and case fatality data for countries where only partial data exist. Rehm et al. (2009) [12] used prevalence, relative risk of mortality, and remission rates to estimate the global incidence of alcohol-use disorders.

Wherever possible, the validity of the DisMod II modelled estimates should be assessed by comparison with actual measured data from the population of interest. This study utilised recent results estimating the incidence of AMI in England using linked hospital episode statistics and death certificates, which captures the vast majority of incident AMIs that occur in England [9] and as such represents a robust dataset for assessment of external validity. The present study complies with all of the ‘best practices’ identified in the ISPOR modelling guidelines for external validation studies [19].

The DisMod II software provides users with a variety of choices about how the input data should be manipulated before the outputs are calculated. These choices include: the method used to interpolate input data to single year estimates (cubic spline or polynomial methods); the shape of the curve to fit the smoothed age-related input data (linear, quadratic, sigmoid, simple exponent or polynomial); and whether or not to allow for time trends in the input data. These three choices alone would generate twenty different sets of results, which we did not choose to display—rather we chose the settings that best suited the epidemiology of AMI. The alternative of using crude input data was not preferable due to inherent problems with the crude data. For example, the prevalence data used in the analyses were taken from the Health Survey for England [7], where rates are reported for 10 year age groups. Using the crude data would lead to large step changes in prevalence as age increases, which resulted in implausible shapes to the modelled incidence data. In practice, changing the specific selections for manipulating the input data did not improve the comparison between the DisMod II estimates and the external dataset. We decided to report the results both for when trends in incidence and case fatality were applied and when they were not as cardiovascular disease rates are decreasing rapidly in the UK and have been for some time [20]. This is important for the DisMod II model, as the method used for solving the differential equations assumes a ‘steady state’ for the disease being modelled (i.e. that age-specific prevalence, incidence and mortality rates for the disease are static). This allows DisMod II to assume that the prevalence of AMI at age t equals the prevalence of AMI at age t-1 plus incident cases minus dying cases. But input data are all taken from the same year (y), whereas data for age t-1 *should* be taken from y-1. This is not a problem if the data in year y-1 are equal to those in year y, but if there are trends in the data (is the case for falling AMI incidence rates), this is not the case. For AMI, the incidence and case fatality rate trend data that we applied were taken from the British Regional Heart Study, a cohort study carried out in British men aged 40–59 at entry between 1978 and 2000 [14]. This study only examines trends in coronary heart disease in men, however other studies report declines of similar magnitude in both men and women [21, 22]. A sensitivity analysis which applied more recent data that were specific to both men and women did not improve the validity of the modelled results. It is not possible to apply age-stratified trends in DisMod II and therefore the same trends were applied across all age groups and the analyses reported here. However, whilst overall there has been a decline in incidence rates, this decline varies by age with the lowest rate of decline occurring in those aged 85 and over [9]. Both the mis-match between the external and modelled datasets and reported declines in AMI incidence are age-specific, making this a likely candidate for the failure of the model to produce externally valid estimates of the incidence of AMI in England. However, without further investigation using a model that can incorporate age-specific trends in incidence and mortality it is not possible to prove this assertion.

Another important limitation of our validity assessment is our input data for mortality. The ideal data for the DisMod II model would be estimates of the increased all-cause mortality rate for people who have previously had a heart attack. We were unable to find direct measures, so we used data on all deaths where AMI was indicated as either the primary cause or a contributing factor. This accounts for the fact that mortality rates from AMI are higher in those that have previously had a heart attack but may not include all increased mortality risk for other conditions (e.g. increased risk of pneumonia) [10, 23].

We found three other studies that compared outputs from the DisMod II software with measured epidemiological data. Manuel et al. (2007) [24] used AMI incidence data from linked hospital records and death certificates data to estimate prevalence of having had an AMI in Ontario, Canada and compared these modelled prevalence rates with estimates derived from a population health survey. The DisMod estimates for both men and women were very similar to those derived from the population health survey, and were within the 95 % confidence intervals. However, the authors did not report on age-specific estimates of prevalence, so it is unclear whether the estimates from the two sources showed similar age trajectories. Saha et al. (2008) [25] compared estimates of prevalence and incidence of schizophrenia derived from DisMod II with paired incidence and prevalence estimates from 15 identified studies. They found that the DisMod II estimates of prevalence were generally higher than those identified in the studies and the estimates of incidence were generally lower, but no age-specific modelled prevalence or incidence rates were reported. One third of the modelled estimates were within 50 % of the estimates from the identified studies. Kruijshaar et al. (2002) [26] compared DisMod estimates of prevalence for breast, prostate, colorectal and stomach cancer with cancer registry data from the Netherlands. Age-specific prevalence estimates were similar to observed rates for colorectal and stomach cancer, but considerably higher for prostate and breast cancer (for some ages modelled estimates were two and three times higher than measured rates, respectively). In all three studies the authors suggested that inadequate description of trends in the studied disease limits the accuracy of the modelled estimates. Given the findings from these studies and the results presented here and the ongoing use of DisMod II in epidemiological modelling studies, it is important that the external validity of DisMod II be further examined with different disease outcomes in different populations.

Another potential source of error in our analyses is the accuracy of the estimates of prevalence of having had AMI from the HSfE. Although the HSfE series is broadly representative of the English population, it does not include residential care home settings in its sample structure. In 2011, around 260,000 people aged 75 and over lived in residential care homes (about 6 % of this age group in England) [27]. Since the incidence rates for AMI are highest in older age groups, this omission may introduce bias for this study. Also, the estimates from the HSfE are based on self report, which could underestimate true rates.

In the absence of measured epidemiological data, the DisMod II model can provide estimates of the incidence of AMI, which may be helpful for health researchers, health care planners and policy makers. Our research suggests that estimates for England may be broadly accurate when applied to the whole population, but can conceal large inaccuracies when studied by age group. One reason for this inaccuracy is the ‘steady state’ assumption of DisMod II and as such the model should be used with caution when estimating the burden of diseases that are changing rapidly within the target population.

## Conclusions

The DisMod II model did not replicate age-specific incidence rates of myocardial infarction observed in the external dataset and therefore did not achieve external validity.
